# The Roles of CircRNAs in Mitochondria

**DOI:** 10.7150/jca.92111

**Published:** 2024-03-17

**Authors:** Donghong Liu, Xinyu Zhou, Yida He, Jun Zhao

**Affiliations:** 1 Department of Special Medical Care, Third Affiliated Hospital of Naval Medical University, Shanghai, 200438, China.; 2 Department of Epidemiology, Naval Medical University, Shanghai, 200433, China.

**Keywords:** mitochondria, mitochondrial genome, noncoding RNAs, circular RNAs, nuclear genome

## Abstract

Mitochondria participate in varieties of cellular events. It is widely accepted that human mitochondrial genome encodes 13 proteins, 2 rRNAs, and 22 tRNAs. Gene variation derived from human nuclear genome cannot completely explain mitochondrial diseases. The advent of high-throughput sequencing coupled with novel bioinformatic analyses decode the complexity of mitochondria-derived transcripts. Recently, circular RNAs (circRNAs) from both human mitochondrial genome and nuclear genome have been found to be located at mitochondria. Studies about the roles and molecular mechanisms underlying trafficking of the nucleus encoded circRNAs to mitochondria and mitochondria encoded circRNAs to the nucleus or cytoplasm in mammals are only beginning to emerge. These circRNAs have been associated with a variety of diseases, especially cancers. Here, we discuss the emerging field of mitochondria-located circRNAs by reviewing their identification, expression patterns, regulatory roles, and functional mechanisms. Mitochondria-located circRNAs have regulatory roles in cellular physiology and pathology. We also highlight future perspectives and challenges in studying mitochondria-located circRNAs, as well as their potential biomedical applications.

## Introduction

**Figure 1:**
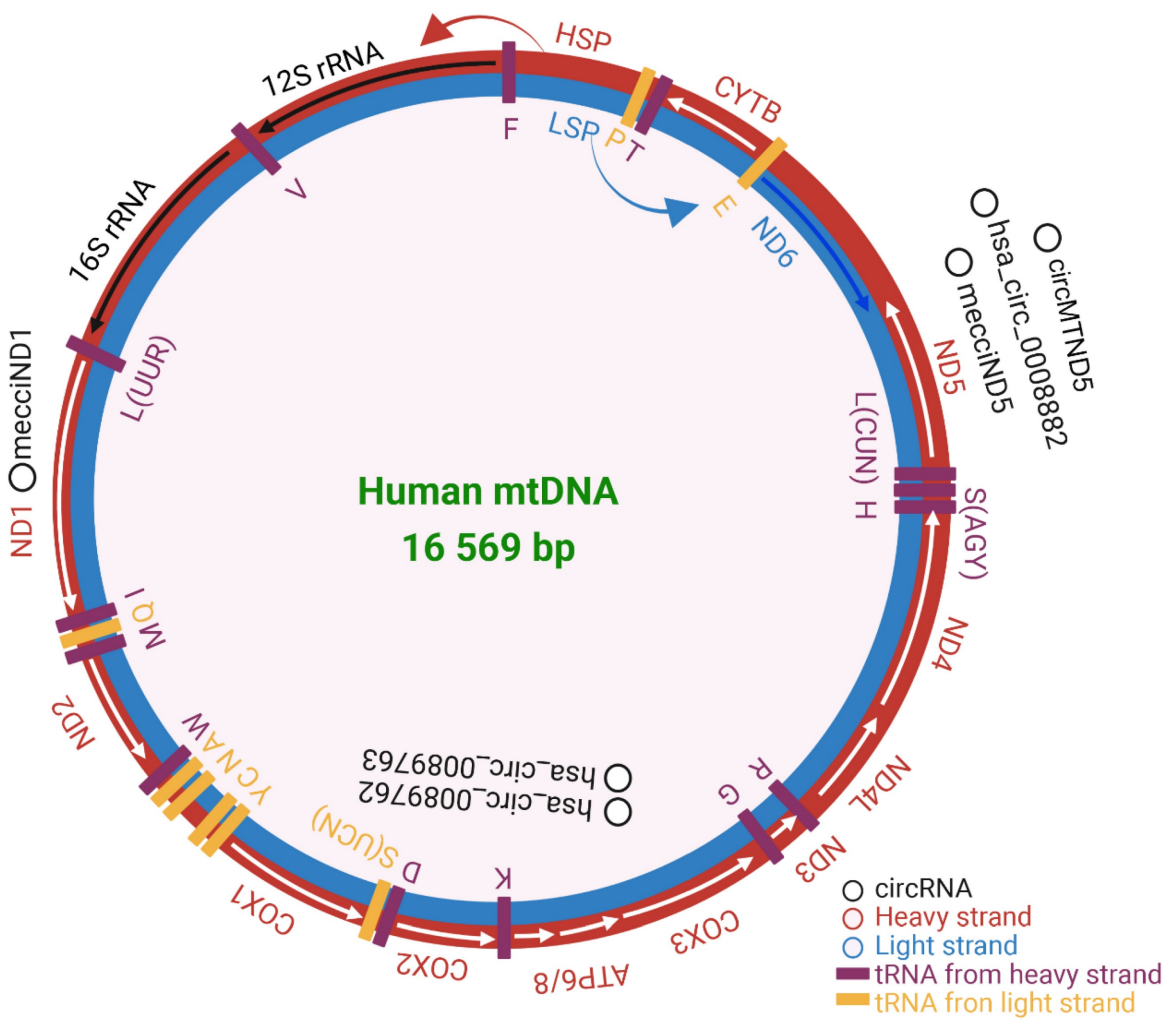
Human mitochondrial DNA composition. Heavy strand in red and light strand in blue. The rRNAs, mRNAs, and tRNAs were separately labeled in black, white, and purple. HSP and LSP are the promoters of heavy and light strands seperately. The validated mecciRNAs were marked in black. The figure was generated by using BioRender (https://app.biorender.com/).

Mitochondria are ancient organelles and are the power factory of cells, producing adenosine triphosphate (ATP) via the electron-transport chain (ETC) and oxidative phosphorylation system (OXPHOS). Mitochondria are involved in many biological functions, such as ATP transport, cell cycle, cell signaling, development, and neuronal function^1^, ^2^. Different from other subcellular organelles, such as Golgi, lysosomes, and endosomes, mitochondria have their own genome. Human mitochondrial genome (mitochondrial DNA, mtDNA) is a double-stranded, 16 569 base pairs (bp) long circular DNA, which is lack of introns and resides within the mitochondrial matrix. Human mtDNA is present in most cells and is conventionally considered to encode 13 protein subunits of the OXPHOS, 2 rRNAs, and 22 tRNAs^3^ (Figure 1). Noncoding RNAs transcribed from the mtDNA are located at the cytosol, nucleus, and plasma^4^. In per diploid cell, the mtDNA copy number is maintained at approximately 1000 to 10 000 copies^5^. The division of mitochondrial genetic information between the nucleus and the mitochondria occurred with gene transfer events during evolution^6^. Gene variation derived from human nuclear genome (nuDNA) cannot completely explain mitochondrial diseases in maternally inherited diabetes and deafness^7^, aging^8^, renal disease^9^, cardiomyopathies^10^, inflammation and immunity^11^, and cancers^12^. This may be resulted from the difference between human mtDNA and nuDNA. Human mtDNA differs from human nuDNA in many aspects, such as non-Mendelian genetics^13^, transcriptional machinery^14^, repair pathways^15^, and the polyploid nature of the genome^16^. Human mtDNAs are packaged into nucleoids. Nucleoids are chromosome-like organellar nuclei^17^, that exhibit a discrete macromolecular assembly that determines mitochondrial genetics and dysfunction^18^ and cardiac homeostasis^19^.

Noncoding RNAs, mainly including long noncoding RNAs (lncRNA), microRNAs (miRNAs), and circular RNAs (circRNAs), play important roles in physiology and pathology^20^, ^21^. CircRNAs are a class of single-stranded noncoding RNAs that are formed in a circular conformation via non-canonical splicing or back-splicing event^22^. CircRNAs were first reported in *Sendai* virus in 1976, and reported as by-products of abnormal splicing^23^, ^24^. The advent of high-throughput sequencing makes circRNAs shine^25^-^27^. Recently, the roles of circRNAs are deeply explored in cancers^28^, immune responses and immune diseases^29^, brain development and central nervous system diseases^30^, and cardiovascular system^31^. Mechanically, circRNAs can regulate transcription, splicing and chromatin interactions, act as miRNA decoys, sequester proteins^27^ and translate proteins, and function as protein scaffolds^32^. CircRNAs are mainly located in the cytoplasm and nucleus^25^, while they are also found in mitochondria^33^. It was briefly reported that 118 mitochondria-located circRNAs were derived from a human cell line HepG2^33^. To classify circRNAs related with the mitochondria clearly, we adopt the term “mecciRNAs” for mitochondrial genome encoded circRNAs which was previously used by Ren BB^34^, and the term “mt-circRNAs” for mitochondria-located circRNAs which was previously used by Liang HX^4^. CircRNAs encoded by nuclear genome were termed “nuc-circRNA”. Mt-circRNAs include both a part of mecciRNAs and a part of nuc-circRNAs. Therefore, circRNAs related with the mitochondria can be classified into three types based on their location and genome origin: circRNAs encoded by mitochondrial genome and located at mitochondria, circRNAs encoded by mitochondrial genome and located at cytoplasm or secreted out of the cells, and circRNAs encoded by nuclear genome and located at mitochondria.

The question arises as to why nuclear genome encoded the same circRNAs are located at mitochondria, despite their crucial roles when located at the cytoplasm and nucleus. Since the discovery of mecciRNAs, their biological roles remain an enigma. The roles of mitochondria-located mecciRNAs are beyond our understanding too. A deeper understanding of mitochondria-located mecciRNAs will bring a new direction. In this review, we highlight the identification, expression patterns, regulatory roles, and functional mechanisms of mt-circRNAs, as well as their potential biomedical applications.

## Historical perspective of mitochondria

Over the past 130 years, the discovery of mitochondrial genome and its roles has gone through the discovery of genetic functions, the discovery of mitochondria DNA, and the discovery and implication of mitochondrial genome. In the 1890s, Richard Altmann first proposed that mitochondria are organelles of eukaryotes, and speculated that mitochondria have genetic autonomy^35^. In Nass MM and Nass S' work, they found mitochondria contain DNA by electron microscope^36^. In the 1960s, the existence of DNA in mitochondria was confirmed and widely accepted. In 1976, Trembath MK and his collogues first completed mapping the genetic and physical map of yeast mtDNA^37^. The human mtDNA sequence currently in use is modified from the portrait of mitochondrial genome by Grivell LA^38^. The studies conducted by Wallace DC's team had opened up a new field of medical research——mitochondrial disease^39^. In 2016, the advance of mitochondrial replacement therapy (MRT) made it possible that mother with a defective mtDNA could give birth to a healthy child^40^. The studies of circRNA blossomed in recent ten years. However, the discovery of circRNA located at mitochondria is just the beginning.

## Mitochondrial function and disfunction

The most famous role of mitochondria is “powerhouse” of cell^41^, ^42^. The OXPHOS system is composed of several multi-protein complexes, which constitute of the electron transport chain within the extensive inner membrane of the mitochondria^43^. Mitochondrial ATP generation is intimately linked through the function of the ETC, and thus efficient measurement of ETC function can provide insight into mechanisms of physiology and disease. A consequence of electron transfer is the generation of reactive oxygen species (ROS)^44^. There are two main antioxidant systems in the mitochondrial matrix, the glutathione and thioredoxin/peroxiredoxin systems, which regulate the concentration and redox species in the organelle^45^. The overproduction of ROS can promote cancer development by inducing genomic instability, modifying gene expression, participating in signaling pathways, and leading to mtDNA and nuDNA mutate^46^.

The other main function of mitochondria is Ca^2+^ dynamics. The uptake of Ca^2+^ by mitochondria was first observed by Slater EC and his colleagues^47^. There are three pathways of Ca^2+^ entry into mitochondrial matrix: mitochondrial calcium uniporter (MCU)^48^, the “rapid mode” (RaM) mechanism^49^, and the mitochondrial ryanodine receptor (RyR)^50^. Ca^2+^ were excluded mainly by the mitochondrial Na^+^/ Ca^2+^/Li^+^ exchanger (NCLX) exchange for sodium (NaC1)^51^ or lithium (LiC1)^52^ or H^+^^53^. However, the capacity for Ca^2+^ of mitochondria is finite. The overload of Ca^2+^ may lead to produce reactive oxygen species and ultimately lead to cell death^54^.

Mitochondria also have metabolism and synthesis roles, such as amino acid^55^ and ascorbate^56^. GOT1 can consume aspartate to transfer electrons into mitochondria, however, upon ETC inhibition, it reverses to generate aspartate in the cytosol. Aspartate supplementation or overexpression of an aspartate transporter allows cells without ETC activity to proliferate^55^. Mitochondria can regenerate ascorbic acid from its oxidized forms, which may help to maintain the vitamin both in mitochondria and in the cytoplasm. This recycling of ascorbic acid is mitochondrial complex III depended^57^.

## Cross-talk between mitochondria and the nucleus

The realization of mitochondrial roles relies on the genetic information both in mtDNA and nuDNA. Proteins that participate in mitochondrial transcription are nuDNA encoded^14^. Proteins encoded by human nuDNA were imported into mitochondria through several pathways, the presequence pathway, the carrier protein pathway, the redox-regulated import pathway, and the β-barrel pathway^58^. The presequence pathway directs proteins to matrix and inner membrane, the carrier protein pathway directs proteins to the inner membrane, the redox-regulated import pathway directs proteins to intermembrane space, and the β-barrel pathway directs proteins to the outer membrane^58^. TOM, TIM23, TIM22, and SAM complexes are embedded in the outer and inner membranes of mitochondria, and participate in protein transport into mitochondria^59^, ^60^. The import of proteins was finely regulated^61^. It is reported that mecciRNAs facilitate the mitochondria entry of nuclear-encoded proteins by serving as molecular chaperones in the folding of imported proteins^62^.

MiRNAs are located at cytosol, nucleus^63^, and exosomes^64^. Besides, miRNAs were found in human mitochondria^65^, ^66^. Blanchette M and his collogues developed a computational tool, miRdup, which can predict the location of miRNAs^67^. Other RNA were also imported into mitochondria, such as tRNA^68^ and pre-miRNAs^69^. The import of nuclear DNA into mitochondria was reviewed by Konstantinov YM^70^ and Verechshagina NA^71^. The import process includes: before cross-membrane translocation, translocation across the mitochondrial outer membrane, and translocation across the mitochondrial inner membrane^72^. Though, it is suggested that PNPASE mediates the import of most lncRNAs and miRNA-378 into mitochondrial matrix, the mitochondrial translocation mechanisms of human RNAs and DNA are largely unclear^73^, ^74^.

Mitochondria also export RNAs^75^ and mtDNA^76^. The miRNAs encoded by mtDNA seem to be located within mitochondria^77^. It is reported that the export of double-stranded RNAs (dsRNAs) into the cytosol is in a PNPASE-dependent manner^78^. The export of mtDNA is related with mitochondrial permeability transition pore (mPTP) and the outer membrane pore formed by VDAC oligomerization^76^.

## Isolation and identification of nuclear-encoded and mitochondria-encoded circRNAs

CircRNAs arising from linear precursor RNAs and the 5′ and 3′ ends were covalently ligated, therefore the traditionally used biochemical and computational approaches for linear RNA studies cannot perfectly fit for the studies of circRNAs. CircRNAs are resistant to degradation by exonucleases, so the 3'-5' exonuclease RNase R is used to improve their circularity as well as enrichment. Xiao MS and his colleagues improved this standard procedure, thus improving the purification efficiency of RNase R^79^. For the annotation of nuclear-encoded circRNA, the pipeline for predicting circRNAs from ribominus sequencing data was applied as mentioned in this work^80^. The start and end positions of circRNAs in the chromosome can be predicted, but the full-length sequences of circRNAs cannot be identified. Hossain MT and his colleagues solved this problem, they presented an R package FcircSEC (Full Length circRNA Sequence Extraction and Classification), which can extract the full-length circRNA sequences based on gene annotation and the output of any circRNA prediction tools^81^. To acquire more information on mecciRNAs, the enrichment of mecciRNAs is needed. Liu X and his colleagues developed a method for mitochondrial RNA isolation to enrich mecciRNAs, so that more information of mecciRNAs could be acquired from RNA-seq, and provide a brief description of the computational method for mecciRNA identification^82^. To identify circRNAs more reliably, the detection tool constantly advancing^83^. It is well known that mRNAs have isogenous. It has been reported that alternative back-splicing also occurs in circRNAs, similar to linear RNAs, generating isogenous circRNAs (ISO circRNAs)^33^, ^84^. A novel algorithm, CIRI-long, is proposed for circRNA characterization and isoform quantification, also identified including 156 mecciRNAs with GT/AG signals^85^.

There are many datasets which can predict circRNAs and their roles or have collected many information of circRNAs which have been validated, such as circBank (http://www.circbank.cn/help.html)^86^, CIRCpedia v2 (http://yang-laboratory.com/circpedia/)^87^, circBase (http://www.circbase.org/)^88^, CSCD (http://gb.whu.edu.cn/CSCD/)^89^, circRNADb (http://reprod.njmu.edu.cn/cgi-bin/circrnadb/circRNADb.php)^90^, CircNet 2.0 (http://circnet.mbc.nctu.edu.tw/)^91^. But there are different naming systems of circRNAs among them, which may lead to confusion of the same circRNAs. But, the annotation of mecciRNA is limited. Until now, there don't have a specific database for mecciRNAs prediction and function annotation.

## Mitochondria-located circRNAs derived from nuclear genome and their roles

Recently, the mechanisms of circRNA biogenesis have been fully elucidated^25^. Exons, introns, and a combination of both can generate circRNAs, which are respectively named ecircRNAs, ciRNAs, and eiciRNAs^25^. The circularization can be mediated by RNA-binding proteins (RBPs) and spliceosome^92^, ^93^, and reverse complementary motifs rely on intron-pairing and lariat-driven circularization^94^, ^95^. After the formation of circRNAs, commonly they are distributed to nucleus and cytoplasm or secreted out of the cells, but recent studies have found that they are also distributed in mitochondria^33^. Maybe this is resulted from the limited proteins or RNAs encoded by mtDNA^3^. Proteins and RNAs derived from mtDNA are limited. To sustain the homeostasis and duplication of mitochondria, proteins and RNAs encoded by the nuDNA are transported into mitochondria^61^. However, less is known about the biological role and molecular mechanism underlying import of circRNA into human mitochondria in contrast with that of proteins, DNA, and miRNAs.

**Figure 2:**
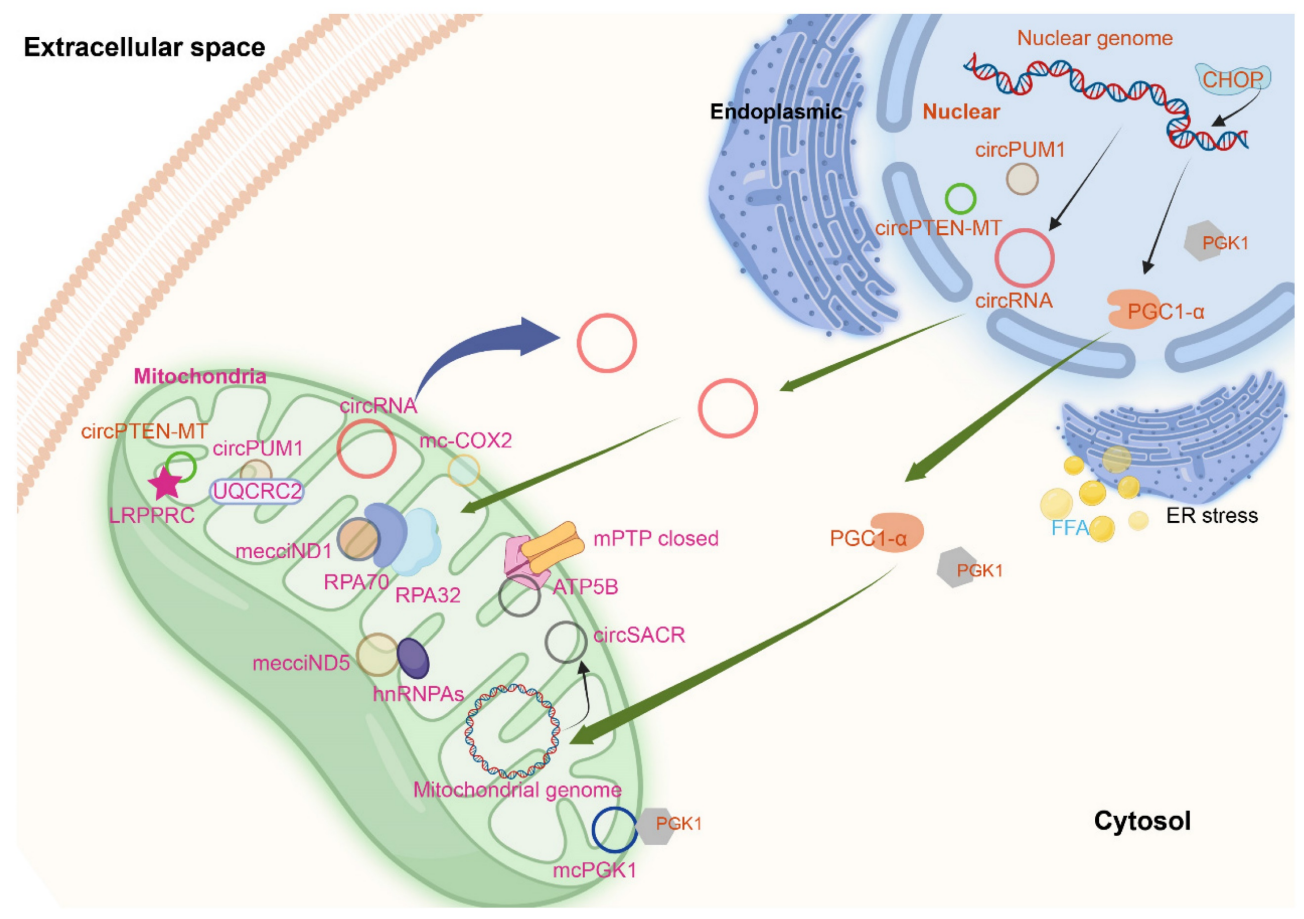
Mitochondria-located circRNAs derived from nuclear and mitochondrial genome. The figure was generated by using BioRender (https://app.biorender.com/).

CircPUM1 is a circRNA generated from the PUM1 gene on human chromosome 1. And multiple confocal assays found that circPUM1 and UQCRC2 co-localized in the mitochondria^2^ (Figure 2). CircPUM1 is positively correlated with HIF1α accumulation under CoCl2-induced intracellular hypoxic-like condition in esophageal squamous cell carcinoma (ESCC) cell lines. Mechanically, circPUM1 acts as a scaffold for the interaction between UQCRC1 and UQCRC2, and circPUM1 depletion induces dysfunction of the mitochondrial complex III and the cleavage of caspase3, thus circPUM1 plays a critical role in maintaining the stability of mitochondrial complex III to enhance oxidative phosphorylation for ATP production of ESCC cells^2^. CircSamd4 was supposed as a biomarker for predicting vascular calcification^96^. CircSamd4 was also found to be mitochondria-located^97^ (Figure 2). CircSamd4 can induce the mitochondrial translocation of the Vcp protein, resulting in the downregulation of Vdac1 and preventing the open of mitochondrial permeability transition pore, thus reducing oxidative stress generation and maintaining mitochondrial dynamics^97^. CircPTEN-MT is another circRNA encoded by nuDNA and located at mitochondria^98^.

There are many circRNAs located outside of mitochondria but can regulate the function of mitochondria, such as circHIPK3^99^, circ-CBFB^100^, circ_0004463^101^, and circFAM160A2^102^. However, it is not reported whether these circRNAs were imported into mitochondria. Exogenous mRNAs, antireplicative RNAs, and single-stranded DNAs were imported into mitochondria^103^-^105^, which occurs without the use of any carriers^58^. The import of proteins into mitochondria is supervised through mitochondrial protein quality control system^106^, however, it is not reported whether the import of circRNAs was supervised or not. Because of the presence of double mitochondrial membranes and the lack of studies about mitochondrial specific circRNA delivery systems, the transfer of nuDNA encoded circRNAs to mitochondria is largely unknown. It is difficult to decode the roles of mitochondria-located circRNAs encoded by nuclear genome now and the regulation of circRNAs import into mitochondria needs further investigation.

## Mitochondria-located circRNAs derived from mitochondrial genome and their roles

The existing hypotheses of circularization mechanisms of circRNAs are mainly based on the studies of nuclear genome derived circRNAs, but the mechanism of mecciRNAs back-splicing has not been proposed until now. Our understanding of the circularization mechanism of mecciRNAs lags behind our knowledge of nuc-circRNAs. MecciRNAs were encoded by both the light and heavy strands of the mtDNA, and the heavy strand of mtDNA encoded the majority^62^. In human HEK cell line, mitochondrial mRNA fragments can be circularized^107^. An earlier study found that in the cells without mitochondria (rho0 MEF cells), no mecciRNAs were identified, whereas mecciRNAs were found in wild-type MEF cells^62^. Despite located at mitochondria, mecciRNAs were also present outside of the mitochondria^62^. In recent years, it has been found that hundreds of mecciRNAs are critical for the adaption of mitochondria to physiological conditions and diseases^62^. The discovery of mecciRNAs may shine a novel light on the communication between mitochondria and the nucleus.

In the plasma samples from chronic lymphocytic leukemia (CLL) patients, 51 circRNAs were remarkably and abnormally expressed. Among the 28 upregulated circRNAs, the top four circRNAs (hsa_circ_0089763, hsa_circ_0008882, hsa_circ_0002363, and hsa_circ_0089762) were all mecciRNAs. Hsa_circ_0089762 is derived by back-splicing from gene COX2, which is located at mtDNA, so termed mc-COX2^108^ (Figure 2). Mc-COX2 is less stable than ciRS-7 and circRPL15 (both circRNAs were derived from nuclear genome), but is much more stable than linear RNAs^108^, ^109^. Mc-COX2 was highly expressed in CLL patients compared with age- and sex-matched healthy persons, and mc-COX2^high^ CLL patients had a worse overall survival (OS) compared with the mc-COX2^low^ group^108^. Functionally, knock down mc-COX2 by siRNAs resulted in a decrease of mitochondrial membrane potential and ATP production, while the proliferation and apoptosis of CLL cells were also regulated^108^. The separate use of crbonyl cyanide 3-chlorophenylhydrazone (CCCP), doxycycline and metformin, can dramatically downregulate the expression of mc-COX2, while the combination of siRNAs against mc-COX2 with CCCP, doxycycline or metformin enhanced the anti-leukemic activity of these drugs^108^.

CircRNA SCAR/has-circ-0089762 (Figure 2), one of the four circRNAs (has-circ-0089736, has-circ-0089762, has-circ-0089763 and has-circ-0008882) derived from mitochondria, was found in liver fibroblasts from patients with nonalcoholic steatohepatitis (NASH). CircRNA SCAR is associated with steatosis-to-NASH progression^109^. Nuclear genome derived circRNAs are produced through a back-splicing mechanism mostly from repetitive elements, such as ALU elements and short intronic repeats (∼30- to 40-nt)^110^. However, different from this, the biogenesis of circRNA SCAR is regulated by hnRNPM, which was verified by siRNAs against hnRNPM and CLIP-seq data. Silencing RNase L increased the circRNA SCAR level in poly(I:C)-treated fibroblasts^109^, ^111^.* In vitro*, circRNA SCAR inhibits mitochondrial ROS (mROS) output and fibroblast activation. PGC-1α mediates circRNA SCAR binding to ATP5B and shuts down mPTP by blocking CypD-mPTP interaction. Furthermore, the effect of PGC-1α is inhibited by lipid overload through ER stress-induced CHOP. Targeting circRNA SCAR *in vivo* alleviates high fat diet-induced cirrhosis and insulin resistance. CircRNA SCAR is one of the molecular components that participate in ER-nucleus-mitochondria-cytosol communication pathway which drives lipid-mediated inflammation^109^.

MecciND1 and mecciND5 are mecciRNAs encoded by the mitochondrial genes ND1 and ND5 respectively, and they are located at both mitochondria and cytosol. RPA70 and RPA32 proteins interact with mecciND1 through TOM40, and the overexpression of mecciND1 results in a higher protein level of RPA70 and RPA32 in the mitochondria, but doesn't change the overall protein level of RPA70 and RPA32. HnRNPA1, hnRNPA2B1, and hnRNPA3 interact with mecciND5. The overexpression of mecciND5 results in a higher protein and mRNA level of hnRNPA1, hnRNPA2B1, and hnRNPA3 in the mitochondria, but the levels of all three hnRNPA proteins and mRNAs were much less affected. These results indicated that mecciRNAs promoted mitochondrial importation of specific protein partners. In hepatocellular carcinoma, the expression of mecciND1 and mecciND5 were upregulated. The use of UV and hydrogen peroxide increased mecciND1 levels, so as to RPA70 and RPA32 protein levels in mitochondria^62^ (Figure 2).

Another circRNA encoded by mitochondrial ND5 gene is circMTND5 (chrM: 14068-14413+)^112^. CircMTND5 sponge MIR6812 and colocalize in mitochondria, alleviating renal mitochondrial injury and kidney fibrosis^112^ (Figure 2). McPGK1 (mitochondrial circRNA for translocating phosphoglycerate kinase 1) is highly expressed in liver tumour-initiating cells (TICs). Its overexpression can drive liver TIC self-renewal^113^. Mitochondria-located circRNAs are listed in Table 1.

## Applications and future aspirations of mt-circRNAs

Mt-circRNA can regulate the energy metabolism of mitochondria. It is reported that the level of PTEN is related with mitochondrial energy metabolism in cell lines^114^. The PTEN expression cells have a lower ATP content and higher ADP/ATP ratio, higher AMPK activating-phosphorylation evoking energy impairment, higher OXPHOS complexes and PGC1α-Sirt3-p53 protein abundance^114^. CircPTEN-MT is a circRNA encoded by exons 3, 4, and 5 of PTEN, which is localized at mitochondria and physically associated with leucine-rich pentatricopeptide repeat-containing protein (LRPPRC)^98^. The downregulation by siRNAs against circPTEN-MT can decrease the mRNA level of the mitochondrial complex Ι subunit and reduce mitochondrial membrane potential and ATP production^98^. It seems that PTEN and circPTEN-MT have an opposite role in regulating mitochondrial energy metabolism. However, these two studies were carried out in different labs and different disease models. The relative expression level and dynamics of PTEN and circPTEN-MT have not been elucidated. Whether PTEN and circPTEN-MT counteract with each other to balance the energy metabolism of mitochondria needs further study. It is reported that mcPGK1 regulates metabolic reprogramming by inhibiting mitochondrial OXPHOS and promoting glycolysis^113^. Those studies indicate that targeting mt-circRNAs has the potential to regulate the energy metabolism of mitochondria and further a potential to regulate the chemoresistance of cancers.

Mt-circRNA can assist the transport of proteins to mitochondria. TOM, TIM23, TIM22, and SAM complexes are embedded in the outer and inner membranes of mitochondria, and participate in protein transport into mitochondria^59^, ^60^. It is reported that mecciRNAs facilitate the mitochondrial entry of nuclear-encoded proteins by serving as molecular chaperones in the folding of imported proteins^62^. McPGK1 can promote PGK1 mitochondrial import via TOM40 interactions at outer mitochondria membrane^113^. The most studied mechanism of circRNA is miRNA sponges and protein scaffolds, however, whether circRNAs can sponge or carry drugs or small-molecule inhibitors to assist the import of them to mitochondria has not been reported. It is worth to devote our efforts to figure out the mystery.

## Discussion

Mitochondrial dysfunction is related with a series of diseases, such as neurodegenerative diseases^115^, cancer^115^, diabetic kidney disease^116^, cardiovascular diseases^117^, and rare diseases^118^. Mitochondrial genome is in small size but highly utilized. The advent of high-throughput sequencing has expanded our understanding of the known complexity of mitochondrial transcriptome. Besides the generally known mRNAs, rRNAs, and tRNAs, mtDNA also encodes a variety of noncoding RNAs such as lncRNAs, sncRNAs, dsRNAs, and circRNAs with diverse regulatory functions. So far, studies of circRNAs encoded by nuDNA have been performed widely, while the roles of mecciRNAs were less understood. MecciRNAs may reside in or shuttle out of the mitochondria and contribute to the nucleus-mitochondria communication, thus posing a difficulty in functional study, such as mecciND1 and mecciND5^62^. Despite the high utilization efficiency of mtDNA, the homeostasis and duplication of mitochondria is resort to proteins and noncoding RNAs encoded by the nuclear genome. CircPUM1 is mitochondria-located circRNA derived from nuDNA. CircPUM1 plays important roles in mitochondria.

Recent studies have brought hints of circRNAs involved in maintaining mitochondrial function, indicating that the circRNAs located at mitochondria are of importance. Nevertheless, many aspects of circRNAs in mitochondria remain unsolved. Firstly, the import processing of nuclear genome encoded circRNAs into mitochondria is not clear. MtDNA encodes limited genes^3^, not only the transcription of mtDNA needs the assistance of nuDNA encoded proteins but also the processing of mitochondrial RNA (mtRNA) needs^119^. NuDNA encoded proteins designated to mitochondria were imported into mitochondrial membrane and mitochondrial matrix by several pathways^58^. CircRNA is the new star of research, the majority of circRNAs encoded by nuclear genome are located at cytoplasm and nucleus^25^. However, recent studies found that in mitochondria there are nuc-circRNAs. But, none of them elucidate the import processing of nuc-circRNAs into mitochondria. Are there any carriers or chaperonins or portholes? Are the import pathways generally applicable or specialized? We need further studies in this field. Secondly, the mechanism of the dynamic regulation of mt-circRNAs has not been identified. CircRNAs are both 5' and 3' end lacking, so the major RNA decay pathways which are often initiated via exoribonucleases are unserviceable to decay circRNAs^120^. Endoribonucleases are enzymes that can cleave RNA without a free 5' or 3' end^121^. It is reported that circRNAs can be degraded by endoribonuclease with the binding of miRNAs and subsequent Ago2^122^. Upon poly(I:C) stimulation or viral infection, circRNAs are globally degraded by endoribonuclease RNase L^123^. Leung AKLand his collogues found that UPF1 and G3BP1 regulate highly-structured circRNAs^124^. GW182 regulate a subset of circRNAs degradation in *Drosophila*, three homologs of GW182-TNRC6A, TNRC6B, and TNRC6C in human control degradation of human circRNAs similarly^125^. These circRNAs decay pathways have not been verified in mitochondria, and it is unknown if there are other mechanisms that can dynamically regulate mt-circRNAs. Deeper investigations are needed to elucidate these questions. Thirdly, it is suggested that nuDNA encoded spliceosomes can mediate the splicing of mtRNA^111^, but it is unknown whether they are participated in regulating the splice of mecciRNAs. There are other questions to be solved, such as the modification of mt-circRNAs, the roles of the identified mecciRNAs, and the functions of mecciRNAs outside mitochondria. Besides, there is a lack of standard nomenclature for circRNAs, which may result in ambiguity in different circRNAs. Such as circRNA SCAR^108^ and mc-COX2^109^ were both named has-circ-0089762. Because of the organelle double membranes, there are still no efficient methods to directly modify the mtDNA *in vivo*. There are some circRNAs that are not located in mitochondria, but can interfere the function of mitochondria, such as circ_0004463^101^, circEZH2^126^, and circFoxO3^127^. The future focuses on isolation, identification, verification, and modification of mecciRNAs will enhance our understanding about the characters and roles of mecciRNAs.

The expression of mt-circRNAs is correlated with mitochondrial function. The aberrant expression of diverse mt-circRNAs has been observed in various cancer cell types, as evidenced by multiple studies. The precise contribution of mt-circRNAs in different cancer development and its clinical implications remain to be elucidated. Further study on the mechanism and clinical significance of mt-circRNAs is conducive to the discovery of new targeted drugs and clinical markers for mitochondria-related diseases, especially cancer.

We are just arrived at the gate of mt-circRNAs, the indoor world landscape of mt-circRNA is far beyond our understanding now. Much work is needed to unveil the circRNA world in mitochondria. Our future studies should concentrate on but not be limited to: the import mechanism of nuc-circRNA into mitochondria and their roles, the genesis of mecciRNA and their function in mitochondria, the dynamics regulation, and the clinical significance of mt-circRNAs.

## Figures and Tables

**Table 1:** Mitochondria-located circRNAs

CircRNA name	Derived genome	Validation	Diseases	Ref.
circPUM1	Nuclear	Yes	Esophageal squamous cell carcinoma	2
circSmad4	Nuclear	Yes	Myocardial infarction	97
circPTEN-MT	Nuclear	Yes	Hepatocellular carcinoma	98
mecciND1, mecciND5	Mitochondrial	Yes, Yes	Hepatocellular carcinoma	62
hsa_circ_0089763, hsa_circ_0008882, hsa_circ_0002363, hsa_circ_0089762	Mitochondrial	No, No, No, Yes	Chronic lymphocytic leukemia	108
has-circ-0089761, has-circ-0089762, has-circ-0089763, has-circ-0008882	Mitochondrial	No, Yes, Yes, Yes	Nonalcoholic steatohepatitis	109
circMTND5	Mitochondrial	Yes	Lupus nephritis	112
mcPGK1	Mitochondrial	Yes	Liver cancer	113

## Abbreviations

ATP: adenosine triphosphate
ETC: electron-transport chain
OXPHOS: oxidative phosphorylation system
mtDNA: mitochondrial genome
nuDNA: nuclear genome
lncRNA: long noncoding RNAs
miRNAs: microRNAs
circRNAs: circular RNAs
mecciRNAs: circRNAs transcribed from the mitochondrial genome
mt-circRNA: mitochondria-located circRNAs
nuc-circRNA: circRNAs transcribed from the nuclear genome
ROS: reactive oxygen species
MCU: mitochondrial calcium uniporter
RyR: mitochondrial ryanodine receptor
NCLX: Na+/ Ca2+/Li+ exchanger
dsRNAs: double-stranded RNAs
mPTP: mitochondrial permeability transition pore
FcircSEC: full length circRNA sequence extraction and classification
ER: endoplasmic reticulum
ISO circRNAs: isogenous circular RNAs
RBPs: RNA-binding proteins
ESCC: esophageal squamous cell carcinoma
CLL: chronic lymphocytic leukemia
CCCP: crbonyl cyanide 3-chlorophenylhydrazone
SCAR: Steatohepatitis-associated circRNA ATP5B regulator
NASH: nonalcoholic steatohepatitis
mROS: mitochondrial ROS
CHOP: C/EBP homologous protein
TICs: tumour-initiating cells
LRPPRC: leucine-rich pentatricopeptide repeat-containing protein
mtRNA: mitochondrial RNA
